# Multimodal echocardiographic techniques in the diagnosis of cardiac tumors: applications and recent advances

**DOI:** 10.3389/fcvm.2026.1750283

**Published:** 2026-06-17

**Authors:** Yuan Li, Yujian Liu

**Affiliations:** 1 Department of Ultrasound, Zigong Fourth People’s Hospital, Zigong, Sichuan, China; 2 Department of Radiology, Zigong First People’s Hospital, Zigong, Sichuan, China

**Keywords:** artificial intelligence, cardiac tumors, contrast-enhanced ultrasound, multimodal echocardiography, speckle-tracking echocardiography, three-dimensional transesophageal echocardiography

## Abstract

Cardiac tumors are exceedingly rare and exhibit marked pathological heterogeneity. Early clinical manifestations are often nonspecific and easily confused with intracardiac thrombi or inflammatory vegetations, posing substantial challenges for timely recognition and clinical management. Imaging plays a pivotal role in both diagnosis and differential diagnosis. Among the available modalities, echocardiography is widely used as the initial evaluation tool due to its noninvasiveness, real-time capability, and repeatability. However, conventional two-dimensional echocardiography is limited by acoustic windows, spatial resolution, and operator dependency, resulting in suboptimal accuracy in assessing tumor vascularity, attachment sites, and benign–malignant differentiation. Recent advances in three-dimensional echocardiography, transesophageal echocardiography (TEE), contrast-enhanced ultrasound (CEUS), and speckle-tracking echocardiography (STE) have enabled a more comprehensive assessment of structural, perfusion, and functional characteristics. These multimodal approaches have demonstrated superior diagnostic performance over traditional 2D imaging in several studies. Despite the promising outlook, current research still faces significant limitations, including nonstandardized imaging and contrast parameters, insufficient cross-vendor consistency of quantitative indices, a lack of externally validated diagnostic thresholds, and a paucity of high-quality, prospective multicenter evidence. This review systematically summarizes the progress of multimodal echocardiography in cardiac tumor diagnosis over the past decade, evaluates the strengths and limitations of each modality, explores the emerging roles of artificial intelligence (AI) and radiomics in quantitative assessment, and proposes future strategies for standardization, intelligent analysis, and cross-modality integration.

## 1 Introduction

Primary cardiac tumors are exceedingly rare, with an estimated incidence of only 0.001%–0.03%. Among benign lesions, myxomas account for the majority, whereas malignant tumors are predominantly angiosarcomas and undifferentiated sarcomas. Secondary cardiac tumors, which occur far more frequently, most commonly originate from lung cancer, breast cancer, and lymphoma through hematogenous dissemination or direct invasion. Because early symptoms are often nonspecific, these tumors are easily misdiagnosed as intracardiac thrombi or infectious vegetations, leading to delayed treatment and adversely affecting clinical outcomes. Accurate early diagnosis is therefore critical (1–4).

Echocardiography remains the first-line imaging modality owing to its noninvasive nature, real-time capability, and excellent repeatability. With the advancement of two-dimensional and three-dimensional transthoracic and transesophageal echocardiography, contrast-enhanced ultrasound (CEUS), speckle-tracking echocardiography (STE), and intracardiac echocardiography (ICE), multimodal ultrasound has demonstrated substantial advantages in characterizing tumor morphology, perfusion patterns, and dynamic functional changes (5–9).

However, several essential limitations persist in current research.

1. Lack of standardized diagnostic criteria: Studies vary widely in pathological classification systems, imaging acquisition parameters, contrast agents, and post-processing algorithms, resulting in substantial heterogeneity (8, 10, 11).
2. Absence of consensus on quantitative indicators: Key metrics—including CEUS perfusion intensity and threshold values from TDI and STE—remain inconsistent across centers (10, 12, 13).
3. Insufficient integration of multimodal information: Most investigations rely on single-modality analysis and fail to achieve a comprehensive quantitative assessment of structural, perfusion, and functional features (8, 14–16).
4. Weakness of the current evidence base: The majority of studies are single-center and retrospective, lacking prospective, multicenter validation or prognostic analysis (8, 10–12, 17).

Given these limitations, this review aims to provide a structured and critical overview of recent advances in multimodal echocardiography for cardiac tumor diagnosis, systematically evaluating the strengths and limitations of each technique while highlighting emerging directions toward standardization, quantitative assessment, and intelligent integration. To enhance methodological transparency and reproducibility, a structured literature search and study selection strategy was applied, as detailed below.

### 2 Literature search strategy and study selection

To ensure methodological transparency and reproducibility, a structured literature search was conducted using PubMed, Web of Science, and Embase databases. Publications from January 2014 to October 2025 were systematically reviewed, with earlier landmark studies included selectively to provide historical and technical context when necessary.

Search strategies combined controlled vocabulary and free-text terms related to cardiac tumor imaging, including “cardiac tumor,” “cardiac mass,” “echocardiography,” “contrast-enhanced ultrasound,” “CEUS,” “speckle-tracking echocardiography,” “three-dimensional echocardiography,” “multimodal imaging,” and “artificial intelligence.” Reference lists of key reviews, consensus statements, and guideline documents were manually screened to identify additional relevant studies.

Priority was given to original investigations, multicenter cohorts, technical reviews, and expert consensus documents addressing echocardiographic characterization of cardiac tumors or multimodal imaging integration. Case reports with limited generalizability, studies lacking detailed imaging methodology, and purely pathological or surgical series without imaging correlation were excluded unless they provided essential illustrative insights.

Given the rarity of cardiac tumors and the methodological heterogeneity of the available literature, this work follows a structured narrative synthesis rather than a formal meta-analysis. Emphasis was placed on methodological trends, diagnostic performance metrics, and current challenges related to quantitative standardization, cross-vendor variability, and multimodal integration.

## 3 Current status of echocardiographic diagnosis of cardiac tumors

### 3.1 Primary cardiac tumors

Primary cardiac tumors are rare but pose substantial diagnostic challenges in cardiovascular imaging. Approximately 75% of these tumors are benign, with myxomas being the most common, followed by papillary fibroelastomas, lipomas, fibromas, and rhabdomyomas (1–4).

Echocardiography is the first-line imaging modality, providing real-time, noninvasive visualization of intracardiac masses, their anatomical location, and associated hemodynamic effects. Two-dimensional transthoracic echocardiography (TTE) remains the foundational assessment method, typically demonstrating pedunculated, highly mobile, and well-circumscribed masses with iso- or hypoechogenicity. Transesophageal echocardiography (TEE), with its higher spatial resolution, provides superior visualization of structures such as the left atrium, valvular attachment points, and pulmonary vein ostia. Three-dimensional TTE/TEE further enables volumetric reconstruction of the tumor and its attachment site, offering precise spatial delineation for preoperative planning (5–9).

**Figure 1:**
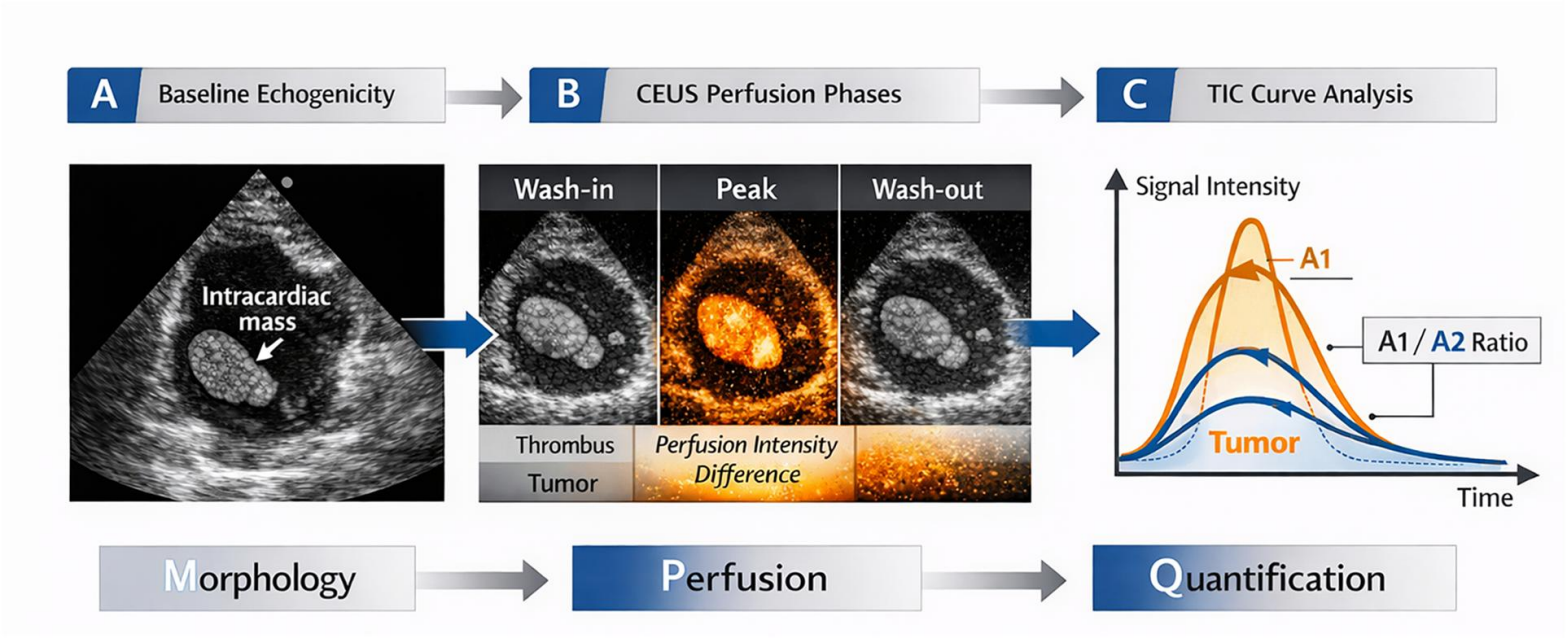
Schematic illustration of contrast-enhanced ultrasound (CEUS) perfusion assessment and time–intensity curve (TIC) modeling in cardiac tumors. **(A)** Baseline echocardiographic visualization demonstrating an intracardiac mass with heterogeneous speckle echogenicity. **(B)** Representative CEUS perfusion phases illustrating wash-in, peak enhancement, and wash-out patterns, highlighting vascularity differences between tumors and thrombi. **(C)** Conceptual TIC analysis demonstrating quantitative perfusion modeling and derivation of indices such as A1/A2. Quantitative parameters are protocol- and vendor-dependent and should be interpreted cautiously rather than as fixed diagnostic thresholds.

In recent years, contrast-enhanced ultrasound (CEUS) has emerged as a powerful tool for quantitative characterization of cardiac tumors. In a prospective multicenter study by Li et al. (10), the CEUS-derived peak intensity ratio (A1/A2)—defined as the ratio of peak enhancement intensity between the tumor and adjacent myocardium—demonstrated excellent diagnostic performance for differentiating cardiac tumors from thrombi. Receiver operating characteristic analysis identified an optimal cutoff value of 0.295, yielding an AUC of 0.958 (10, 12). Nevertheless, this cutoff represents a statistically derived threshold obtained within a specific acquisition protocol and ultrasound platform rather than a universally applicable clinical standard. Subsequent investigations, including the work by Yang et al. (12), further highlighted the biological relevance of CEUS perfusion parameters by demonstrating correlations with immunohistochemical markers such as Ki-67 and CD31, suggesting that these indices primarily reflect tumor vascularity and microvascular density (12). Importantly, current technical reviews and consensus statements emphasize that quantitative CEUS metrics remain highly vendor- and software-dependent, and no cross-vendor validated cutoff has been established to date. Therefore, numerical thresholds such as A1/A2 should be interpreted as exploratory findings that require standardized acquisition and multicenter validation before clinical generalization (8–12) (Figure 1). For example, CEUS time–intensity curve–derived parameters may vary substantially across ultrasound platforms because of differences in contrast-specific presets, mechanical index, dynamic range, region-of-interest placement, and proprietary curve-fitting algorithms, limiting the direct transferability of quantitative values between vendors (1, 8, 12–15).

Recent reviews highlight that integrating TTE/TEE with cardiac magnetic resonance (CMR) or computed tomography (CT) compensates for the limitations of single-modality imaging and significantly enhances diagnostic accuracy, particularly in infiltrative or multifocal disease (5, 8, 18, 19). Overall, multimodal echocardiography has evolved from simple morphological description to a more sophisticated quantitative and functional assessment framework centered on vascularity, perfusion metrics, and dynamic kinematic features (8, 20, 21).

From a practical clinical perspective, the integration of advanced echocardiographic techniques into routine workflows remains an important consideration. In most centers, contrast-enhanced ultrasound is performed by experienced sonographers or cardiologists following standard transthoracic or transesophageal examinations, typically adding only several minutes to the acquisition time while providing incremental perfusion information. However, quantitative CEUS analysis and strain-based assessment require dedicated training, familiarity with post-processing software, and awareness of vendor-specific variability. Rather than replacing conventional imaging pathways, multimodal echocardiography is increasingly used as a stepwise strategy—serving as a first-line, radiation-free tool for rapid characterization, with cardiac magnetic resonance or computed tomography reserved for complex or equivocal cases. Future standardization of acquisition protocols and reporting criteria will be essential to facilitate broader clinical adoption.

Despite promising results, interpretation of CEUS-derived quantitative indices requires caution. Most available studies are limited by small sample sizes—often fewer than 60 cases—and thresholds are frequently derived from single-center cohorts. Device dependency, variability in acquisition protocols, and operator-related factors remain major sources of interstudy heterogeneity, restricting external reproducibility and wider clinical adoption (8, 10, 12, 22). Furthermore, the absence of standardized protocols for contrast administration, imaging acquisition, and post-processing algorithms contributes to substantial variability in quantitative measurements (10, 11, 23). The predominance of retrospective designs, together with the lack of prospective multicenter validation, further limits the strength of current evidence. Multiple quantitative parameters—including A1/A2, TPI/MPI, and AUTIC—have yet to reach consensus thresholds, while ultrasound–pathology correlation studies remain scarce, hindering a deeper understanding of the biological basis of perfusion patterns. In addition, insufficient control of vendor-dependent settings, frame rates, and mechanical index (MI) continues to exacerbate interstudy variability (10–12, 15, 23, 24).

Artificial intelligence (AI) and radiomics are increasingly explored for automated segmentation and malignancy prediction of cardiac tumors; however, their current clinical role remains supportive rather than definitive, owing to persistent challenges in data standardization and model interpretability (25–27). Inter-observer reproducibility metrics, including intraclass correlation coefficients (ICC) and *κ* statistics, remain inconsistently reported across both manual and AI-assisted echocardiographic analyses, representing an important methodological gap that limits cross-study comparability and clinical translation (24).

AI-assisted tools may facilitate workflow standardization and provide preliminary guidance, but expert human validation remains essential. This consideration is particularly relevant in anatomically complex regions, where physiological structures such as Eustachian valves, Chiari networks, and prominent trabeculations can mimic tumor-like features and introduce potential pitfalls for automated algorithms (7, 14). Beyond the well-recognized “black-box” nature of deep learning models, additional technical and practical challenges persist, including variability in acquisition protocols, cross-vendor differences in strain and CEUS quantification, limited availability of large, well-annotated datasets for rare cardiac tumors, and difficulty distinguishing true pathological enhancement from artifacts or motion-related noise (25–27). Moreover, many current AI models lack robust external validation and may show limited generalizability across institutions with heterogeneous imaging practices. Accordingly, AI-based analysis should be regarded as an adjunctive decision-support tool rather than a substitute for experienced clinical judgment (25, 27).

### 3.2 Secondary cardiac tumors

Secondary cardiac tumors are far more common than primary tumors, with an incidence estimated to be 20–40 times higher. They most frequently originate from lung cancer, breast cancer, renal cell carcinoma, melanoma, and lymphoma, reaching the heart through hematogenous spread, lymphatic dissemination, or direct invasion. Clinical manifestations are typically nonspecific, and many cases are incidentally detected due to pericardial effusion, arrhythmias, or heart failure (8, 28, 29).

Two-dimensional transthoracic echocardiography (TTE) enables dynamic evaluation of pericardial, intracavitary, and significant vessel involvement. Typical findings include hypoechoic masses in the right atrium or right ventricle, tumor thrombi extending into the vena cava, and pericardial effusion with floating echogenic material. Transesophageal echocardiography (TEE) provides superior visualization of lesions on the posterior left atrial wall, the pulmonary vein ostia, and basal cardiac structures, enabling more accurate delineation of attachment sites and the depth of infiltration (8, 20).

Color Doppler ultrasound (CDUS) can highlight differences in tumor vascularity: hypervascular metastases (such as those from melanoma or renal cell carcinoma) exhibit abundant flow signals, whereas hypovascular metastases (such as those from breast cancer) show reduced perfusion (18, 28). Contrast-enhanced ultrasound (CEUS) further improves characterization by clearly distinguishing tumors from thrombi, effusions, or inflammatory tissue. Malignant lesions typically display rapid enhancement followed by delayed washout, whereas thrombi show no enhancement (10–12).

Several studies have reported that CEUS achieves an area under the curve (AUC) exceeding 0.95 in differentiating metastatic tumors from thrombi, and multimodal imaging integration substantially enhances diagnostic accuracy (10–12). Evidence also indicates that cardiac lesions demonstrating markedly increased metabolic activity on FDG-PET/CT or PET/MR often correspond to hyperperfused, potentially malignant regions depicted by CEUS, reflecting the physiological correlation between metabolic demand and vascular supply. Multimodal integration not only improves the identification of malignant features but also helps select optimal biopsy targets, thereby increasing overall diagnostic accuracy and confidence—particularly in complex or diagnostically challenging cases (8, 30–33).

Although no consensus quantitative CEUS thresholds have been established for secondary cardiac tumors, qualitative perfusion characteristics may still provide clinically meaningful insights when interpreted in conjunction with tumor biology and clinical context. Contrast-enhanced ultrasound primarily reflects lesion microvascularization, and differences in vascular architecture among primary malignancies may influence enhancement patterns. In this context, cardiac metastases from renal cell carcinoma and melanoma—tumors characterized by prominent angiogenesis—may demonstrate relatively strong or early enhancement, reflecting their hypervascular biological behavior. By contrast, metastases originating from breast cancer and most lung carcinomas, which represent the most common sources of secondary cardiac tumors, more often exhibit weaker, delayed, or heterogeneous enhancement patterns consistent with comparatively lower microvascular density. These qualitative enhancement features should not be interpreted as tumor-specific diagnostic criteria; rather, they serve as adjunctive indicators that may help narrow differential diagnoses and guide further multimodal evaluation, particularly when integrated with clinical history and complementary cross-sectional imaging such as CMR or PET/MR.

Despite the critical role of multimodal ultrasound in evaluating secondary cardiac tumors, several significant limitations persist in current research. First, most available studies are case series or single-center analyses, with limited large-scale prospective validation. Moreover, considerable variability exists in vascular patterns and echocardiographic appearances across different primary malignancies, making it challenging to establish unified diagnostic criteria. Second, although quantitative CEUS parameters such as A1/A2 and TPI/MPI have been partially validated for primary tumors, no consensus thresholds have been established for secondary tumors. Third, cross-modality correlation studies remain scarce. While PET/MR provides metabolic information, ultrasound excels at assessing dynamic blood flow and valvular function, yet standardized algorithms integrating metabolic markers with ultrasound-derived quantitative parameters are lacking. Accurate multimodal evaluation should occur at the data-integration level rather than through simple side-by-side comparison. Fourth, the 2023 European Association for Cardiovascular Imaging (EACVI) consensus highlights the absence of evidence-based guidelines for metastatic cardiac lesions, particularly regarding the differentiation between tumor thrombi and metastatic masses, the assessment of pericardial involvement, and postoperative therapy monitoring (10, 11, 25, 34, 35).

Future research should focus on several key directions (2, 8, 31):

1. Developing cross-modality databases to enable multidimensional validation across CEUS, PET/MR, and CMR;
2. Building AI-driven vascular-pattern recognition models to facilitate early detection of micro-metastatic disease;
3. Establishing standardized, cancer-specific reporting systems (e.g., tailored templates for lung cancer, breast cancer, or renal cell carcinoma metastases);
4. Exploring radiomics-based perfusion and time–enhancement curve texture features for prognostic prediction.

In summary, multimodal ultrasound is transitioning from a purely observational tool toward an integrated functional–molecular diagnostic approach. Its synergistic application with PET/MR is expected to become a cornerstone in the future management of cardiac metastases.

## 4 Comparison of multimodal echocardiographic techniques: advantages and limitations

With the continued advancement of transthoracic echocardiography (TTE), transesophageal echocardiography (TEE), three-dimensional echocardiography (3D echo), color Doppler ultrasound (CDUS), contrast-enhanced ultrasound (CEUS), and tissue Doppler/strain imaging (TDI/STE), the echocardiographic evaluation of cardiac tumors has progressed from traditional structural description to a multidimensional quantitative framework integrating function, perfusion, and spatial characterization (8, 23, 36).

Recent reviews and expert consensus statements consistently emphasize that echocardiography is no longer limited to assessing tumor size, morphology, and mobility. Increasing attention is now placed on perfusion characteristics, vascular patterns, regional myocardial functional impairment, and the role of cross-modality integration (7, 14, 37–40).

**Figure 2:**
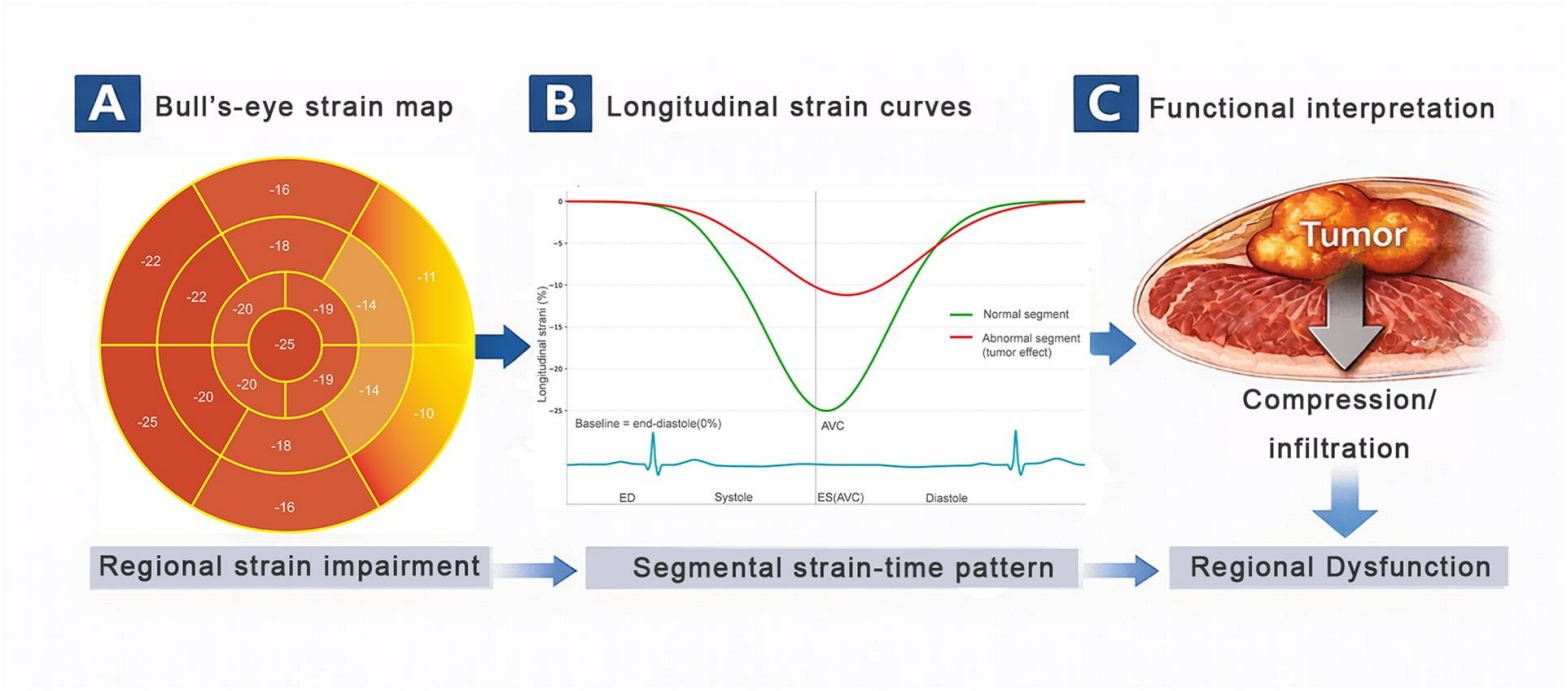
Schematic illustration of speckle-tracking echocardiography (STE)–based functional assessment in cardiac tumor evaluation. **(A)** Simplified bull's-eye map demonstrating regional longitudinal strain distribution, highlighting localized reduction in myocardial deformation adjacent to a cardiac mass. **(B)** Representative strain curves illustrating altered myocardial deformation patterns in tumor-adjacent segments compared with normal myocardium. **(C)** Conceptual framework showing how tumor-related compression or myocardial infiltration may lead to regional functional impairment detectable by STE. This schematic emphasizes the role of STE as a complementary functional modality that provides quantitative insights beyond structural imaging, while acknowledging that strain measurements remain dependent on image quality, acquisition protocols, and vendor-specific post-processing algorithms.

Accordingly, Table 1 provides a systematic summary of the core value and clinical positioning of each echocardiographic modality. TTE remains the foundation of initial screening and is essential for evaluating tumor burden and hemodynamic consequences. TEE provides irreplaceable high-resolution visualization of the left atrial posterior wall, valvular attachment points, and pulmonary vein ostia. Three-dimensional echocardiography is superior to two-dimensional imaging for assessing complex tumor attachment sites and for preoperative spatial planning. CDUS plays a crucial role in identifying flow alterations associated with tumor invasion. CEUS, supported by multicenter evidence, currently has the most significant quantitative potential—its perfusion parameters (e.g., A1/A2, TPI/MPI) demonstrate excellent diagnostic performance (AUC > 0.90) in differentiating thrombi from tumors and distinguishing benign from malignant lesions. Additionally, TDI/STE enables early detection of localized myocardial dysfunction due to tumor compression or infiltration, providing functional insights beyond structural assessment (Figure 2).

**Table 1:** Comparison of multimodal ultrasonic techniques in cardiac tumor evaluation.

Modality	Major Advantages	Typical Clinical Applications	Key Limitations	Evidence & Diagnostic Value
TTE	Bedside availability; real-time hemodynamic assessment	Initial screening; tumor size/mobility; LVOT obstruction	Limited by acoustic window; poor visualization of posterior structures	Sensitivity 75%–85%; >90% when combined with CEUS
TEE	High resolution; excellent visualization of posterior structures and pulmonary veins	Hidden lesions; pre-operative localization	Semi-invasive; patient tolerance and cost limitations	Superior for left atrial posterior wall and valve attachment assessment
3D TTE/TEE	Accurate 3D spatial relationships; volumetric measurement	Complex tumors; valvular attachment lesions	Time-consuming processing; strongly operator- and quality-dependent	Improves anatomical localization; useful for surgical planning
CDUS	Dynamic hemodynamic evaluation	Assess stenosis/regurgitation; perfusion pattern differences	Insensitive to low-velocity flow; angle-dependent	Supports benign-malignant differentiation but not diagnostic alone
CEUS	Perfusion imaging; quantitative differentiation of benign vs. malignant	Thrombus vs. tumor; perfusion characterization	Non-standardized TIC models; device-dependent variability	High diagnostic performance (AUC >0.90); vendor-dependent quantitative metrics with exploratory, non-standardized thresholds
TDI/STE	Quantitative local myocardial function assessment	Tumor compression/infiltration-related regional dysfunction	TDI: angle-dependent; STE: relatively angle-independent but sensitive to image quality, frame rate, and rhythm.	Detects early impairment; current evidence limited

This table summarizes the major advantages, clinical applications, limitations, and diagnostic value of different ultrasound modalities in cardiac tumor assessment.

To facilitate cross-study comparison and provide a more objective context for modality selection, representative diagnostic performance data from key studies are summarized in Table 2.

**Table 2:** Representative studies reporting diagnostic performance of multimodal imaging techniques for cardiac masses.

**Study**	**Design/population**	**Modality**	**Diagnostic task**	**Key parameter(s)**	**AUC**	**Sensitivity**	**Specificity**	**Key message/limitation**
Li et al. (10)	Prospective multicenter cross-sectional study; 108 patients with suspected cardiac masses	CEUS	Tumor vs. thrombus	A1/A2 cutoff 0.295	0.958	100.0%	91.7%	Strong multicenter evidence for CEUS in tumor-thrombus differentiation
	Same cohort; 30 benign vs. 36 malignant tumors	CEUS	Malignant vs. benign tumor	A1/A2 cutoff 1.28	0.886	80.6%	96.7%	Quantitative threshold useful, but less robust than combined CE assessment
	Same cohort	CEUS	Malignant vs. benign tumor	Combined qualitative + quantitative CE analysis	0.953	97.2%	93.3%	Combined CE interpretation outperformed single-parameter assessment
Wang et al. (11)	Prospective multicenter study; 145 patients	CEUS	Tumor vs. thrombus	A1/A2 cutoff 0.499	0.977	97.9%	88.4%	Confirms high diagnostic value of CEUS in a larger multicenter cohort
	Same cohort; 66 benign vs. 30 malignant tumors	CEUS	Malignant vs. benign tumor	A1/A2 cutoff 1.583	0.950	93.3%	93.9%	Good discrimination between benign and malignant tumors
Yang et al. (12)	Pathology-confirmed cohort; 44 tumors (34 benign, 10 malignant)	CEE	Malignant vs. benign tumor	TPI/MPI	0.850	60.0%	100.0%	CEE perfusion parameters were diagnostically useful and correlated with pathology markers; TPI/MPI showed high specificity.
Paolisso et al. (36)	Observational cohort; 286 patients with suspected cardiac masses	Echocardiographic markers	Malignancy prediction	Unweighted score >3; CTA algorithm	>0.90	94.0%	80.0%	Six echo features predicted malignancy; CTA correctly classified 87.5% of malignant masses
Paolisso et al. (9)	Observational cohort; 167 patients with echo + CMR within 1 month	CMR	Malignant vs. benign cardiac mass	CMR mass score cutoff **≥** 5	0.976	NR	NR	Excellent accuracy, superior to echocardiographic mass score
Son et al. (17)	Retrospective study; 41 patients (22 tumors, 21 thrombi)	CMR radiomics/native T1 mapping	Tumor vs. thrombus	Rad score	0.980	95.4%	95.2%	Native T1 radiomics outperformed mean T1 and LGE ratio
Lee et al. (15)	Retrospective multicenter study; training set 192, validation set 63	CT radiomics	Tumor vs. thrombus	Combined model (clinical + conventional CT + radiomics)	0.983 (training); 0.911 (validation)	94.6%; 100.0%	92.3%; 81.3%	Combined CT model outperformed clinical model alone
Bao et al. (26)	Retrospective echocardiography-based ML study; 215 clips from 121 cancer patients	Echo-based radiomics nomogram	Benign vs. malignant classification in cancer patients	Integrated model test cohort)	0.861	96.0%	40.5%	Comparable to senior physician and superior to junior physician
Rizzo et al. (30)	Systematic review and dual meta-analysis; 15 studies, 1,114 patients	[18F] FDG PET/CT	Malignant vs. benign cardiac mass	PET metabolic assessment/SUVmax	NR	89.2%	82.8%	Robust pooled PET performance; malignant lesions had substantially higher SUVmax

AUC, area under the curve; CEUS, contrast-enhanced ultrasound; CEE, contrast-enhanced echocardiography; CMR, cardiac magnetic resonance; CTA, classification tree analysis; NR, not reported; TPI/MPI, tumor peak intensity/myocardium peak intensity ratio.

Diagnostic metrics should be interpreted cautiously because study design, patient composition, reference standards, acquisition protocols, and post-processing methods varied across studies.

While Table 1 summarizes the relative strengths of individual echocardiographic modalities, their clinical value ultimately depends on how they are incorporated into a stepwise diagnostic strategy. In practice, multimodal echocardiography serves as a first-line platform for lesion detection, early phenotyping, and hemodynamic assessment, helping determine which patients can remain on an echo-dominant pathway and which require escalation to second-line imaging (8, 18, 20, 33).

**Figure 3:**
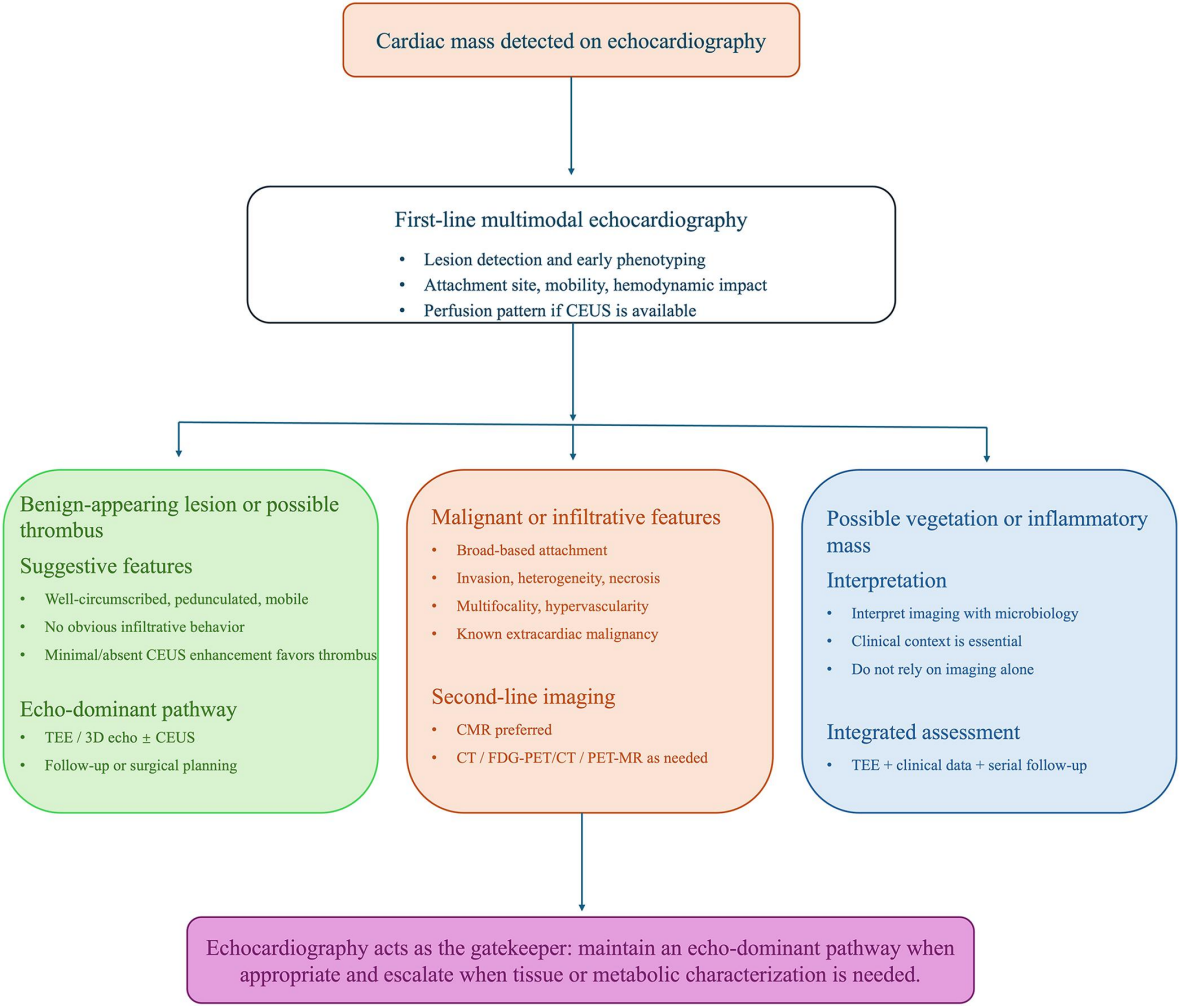
Practical stepwise imaging pathway for cardiac masses. Echocardiography serves as the first-line gatekeeper for lesion detection, phenotyping, and triage. Depending on the dominant imaging scenario, patients may remain on an echo-dominant pathway, undergo escalation to second-line imaging for suspected malignant or infiltrative disease, or require integrated interpretation with clinical and microbiological data in cases of possible vegetation or inflammatory mass.

Benign-appearing lesions and masses with absent or minimal CEUS enhancement suggesting thrombus may often be managed within an echo-dominant pathway, supplemented by TEE, three-dimensional echocardiography, and CEUS as appropriate. By contrast, malignant or infiltrative features should prompt escalation to second-line imaging, with CMR preferred for tissue characterization and CT or PET-based imaging used selectively for anatomical extension, staging, or biopsy planning (41). Suspected vegetations or inflammatory masses require integrated interpretation with microbiological, clinical, and serial follow-up data rather than imaging alone (5–12, 18, 21, 30, 31, 33, 35, 36). A practical stepwise pathway summarizing these scenarios is shown in Figure 3.

## 5 Advances in artificial intelligence and radiomics for ultrasound-based diagnosis of cardiac tumors

In recent years, artificial intelligence (AI) and radiomics have shown increasing potential in cardiac imaging, creating new opportunities for more quantitative and reproducible assessment in echocardiography, a modality traditionally dependent on operator experience. Conventional ultrasound interpretation still relies heavily on subjective evaluation of echogenicity, mass morphology, mobility, and flow characteristics, making diagnostic performance vulnerable to inter-operator variability and limiting reproducibility. In this context, AI-based detection, segmentation, and feature-extraction models may help automatically localize cardiac masses on raw B-mode images or CEUS time-series data, delineate lesion boundaries, analyze dynamic perfusion patterns, and quantify imaging features, thereby improving the objectivity of ultrasound as a more data-driven modality for cardiac tumor evaluation (27, 42, 43).

Although the available evidence remains limited, several cardiac-specific studies have already provided proof of concept for quantitative image analysis in cardiac masses. Radiomics features derived from CT and CMR have shown the ability to distinguish cardiac tumors from thrombi, while echocardiography-based predictive models and nomogram approaches suggest that structured quantitative analysis may support malignancy risk stratification and first-line triage in patients with suspected cardiac tumors (15, 17, 25, 26). Indirect methodological support also comes from other tumor types, in which CEUS-derived perfusion parameters and ultrasound-based radiomics features have been associated with aggressive biological behavior and proliferation markers such as Ki-67. However, such evidence should be regarded as hypothesis-generating rather than directly transferable, because cardiac tumors differ substantially from extracardiac solid tumors in rarity, anatomical environment, motion characteristics, and imaging acquisition conditions (25, 44, 45).

From a clinical perspective, the most realistic near-term role of AI in cardiac tumor imaging is likely to be decision support rather than autonomous diagnosis. Potential applications include automated or semiautomated lesion segmentation, extraction of simple quantitative indices from CEUS or strain imaging, identification of predefined malignant red flags, and structured reporting support to reduce omission of key imaging features. In parallel, deep learning algorithms have also demonstrated the ability to guide inexperienced operators in real time to acquire diagnostically adequate echocardiographic views at the bedside, potentially improving consistency in image acquisition and preliminary screening in resource-limited environments (8, 46).

Despite this promise, several major bottlenecks remain. First, most current models are trained on single-center datasets with relatively small sample sizes and lack robust external validation across devices, regions, or probe types. This limitation is especially pronounced in cardiac tumor imaging because of the rarity of the disease and the difficulty of assembling large, well-annotated datasets. Second, echocardiography-specific technical variability remains a major barrier. Unlike CT or MRI, ultrasound image quality is strongly influenced by acoustic window conditions, operator experience, probe orientation, frame rate, and patient-related motion, all of which constrain feature stability and reproducibility (46–48). Third, inconsistent annotation standards, differences in ROI delineation, TIC fitting methods, and cardiac-cycle selection introduce batch effects that hinder reproducibility across datasets (49, 50). Fourth, interpretability remains insufficient for many deep learning models, while cross-modal fusion is still methodologically immature because ultrasound, CMR, and PET are based on different physical principles and are often integrated without rigorous feature alignment or a shared latent space (51–54).

Future advancements are expected to focus on three major areas (49, 55–57):

1. standardization and enhancement of feature stability through multi-device, multicenter CEUS, TDI, and 3D ultrasound datasets and unified acquisition and feature-extraction workflows;
2. AI-enhanced and interpretable diagnostic models that provide transparent decision-support cues and improve clinical acceptance;
3. clinically oriented integration and decision support, in which echocardiographic structural, perfusion, and functional information is combined with selected cross-sectional imaging features to improve risk triage and follow-up stratification rather than pursue fully autonomous diagnosis.

Until these technologies mature and undergo multicenter validation, AI should be regarded primarily as an assistive tool in the ultrasound evaluation of cardiac tumors rather than a replacement for expert interpretation.

## 6 Multimodal integration and future perspectives

With the continued advancement of imaging technologies, multimodal integration has become increasingly central to the diagnostic evaluation of cardiac tumors. Echocardiography remains the first-line imaging modality because of its real-time capability, dynamic assessment, broad accessibility, and usefulness in evaluating lesion mobility, attachment, valvular involvement, and hemodynamic consequences. Nevertheless, its performance is limited by acoustic window constraints, operator dependency, and a comparatively lower capability for tissue characterization, highlighting the need for complementary cross-sectional and metabolic imaging in selected clinical scenarios.

From a broader modality-agnostic perspective, no single imaging technique can answer all clinically relevant questions in cardiac mass evaluation. CMR provides superior noninvasive tissue characterization and is particularly valuable for assessing myocardial or pericardial infiltration, internal composition, and local extension. CT offers complementary strengths in depicting calcification, extracardiac extension, and detailed anatomic relationships, especially when surgical planning is required or when CMR is contraindicated. FDG-PET/CT or PET/MR adds metabolic information and is most useful in selected patients with suspected malignancy, metastatic burden, or a need for biopsy targeting and systemic staging. Accordingly, the role of multimodal echocardiography should be viewed as complementary and triage-oriented rather than comprehensive in isolation, with each modality contributing distinct and clinically relevant information according to the diagnostic context (8, 18, 30, 31, 33, 35, 58, 59). In practical terms, echocardiography has clear advantages in accessibility, bedside applicability, and lower resource utilization, whereas CMR and PET-based imaging are better reserved for problem-solving scenarios in which tissue characterization, metabolic assessment, or broader oncologic staging is required.

In recent years, the incorporation of multiple echocardiographic submodalities—including 2D and 3D echocardiography, CDUS, CEUS, and TDI/STE—has enabled a more comprehensive “structure-perfusion-function” analytical framework. A 2024 JACC: CardioOncology review by Angeli et al. proposed an “echo-first, modality-verified” diagnostic pathway, in which TTE/TEE and CEUS serve as frontline tools for initial screening and phenotyping, while CMR or PET/MR is selectively introduced when suspicious features require further tissue characterization or metabolic assessment (8). This stepwise use of imaging not only improves diagnostic confidence but may also reduce unnecessary escalation to more costly or less available modalities.

Recent studies have shown that FDG-PET/CT and PET/MR achieve high diagnostic accuracy in differentiating benign from malignant cardiac masses, with metabolic parameters such as SUVmax correlating strongly with malignancy and multicenter studies reporting AUCs typically exceeding 0.85–0.90. Hypermetabolic regions often correspond to enhancing tumor components on contrast-enhanced CT or CMR, suggesting physiologic complementarity between metabolic and perfusion information. However, systematic investigations evaluating the spatial concordance between CEUS perfusion parameters and FDG uptake remain scarce, and this relationship warrants further study (10, 11, 30, 31).

Bodega et al. further proposed a tiered multimodal fusion framework in which echocardiography functions as the initial platform for lesion detection and preliminary phenotyping, followed by targeted integration of CMR markers (e.g., LGE, T1 mapping) and PET/MR metabolic indicators to refine risk stratification, guide surgical planning, and optimize biopsy targeting. Although supporting evidence from other disease domains suggests that multimodal radiomics and AI-based joint modeling can improve classification performance, dedicated multimodal AI research specifically targeting cardiac masses remains very limited. At present, these approaches should therefore be regarded as promising future directions rather than established clinical tools (9, 59).

Future research should prioritize several key directions:

1. establishing unified quantitative frameworks and standardized workflows across devices and centers to enable reproducible CEUS-CMR-PET/MR assessment (55, 60);
2. developing AI-driven fusion models that integrate structural, functional, perfusion, and metabolic features in clinically interpretable ways (61, 62);
3. advancing real-time fusion imaging technologies for intraoperative navigation, tumor-margin evaluation, and dynamic risk-adjusted follow-up (63, 64); and
4. exploring “cardiac tumor digital twin” frameworks that integrate imaging, clinical biomarkers, and treatment-response data to support personalized therapeutic strategies (65, 66).

**Figure 4:**
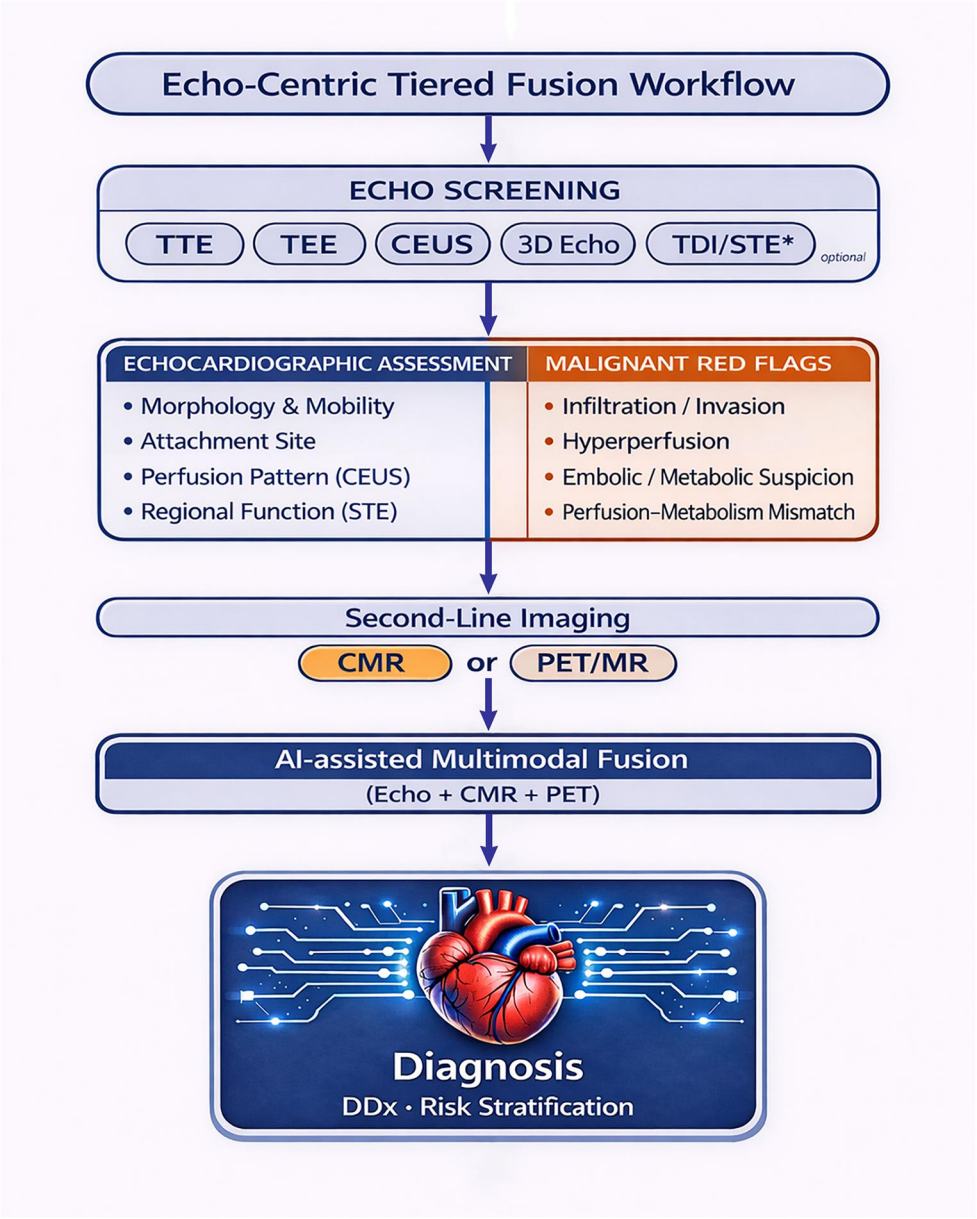
Echo-centric tiered multimodal imaging workflow for cardiac tumor evaluation. Echocardiography serves as the first-line diagnostic platform, integrating structural, perfusion, and functional assessment to identify imaging features suggestive of malignancy. When suspicious findings are present, second-line modalities—including cardiac magnetic resonance (CMR) for tissue characterization and PET/MR for metabolic evaluation—are selectively incorporated. AI-assisted multimodal fusion represents an emerging supportive approach for integrating echocardiography, CMR, and PET-derived information, with the potential to enhance quantitative assessment, differential diagnosis, and risk stratification. This schematic illustrates a tiered, decision-driven imaging strategy rather than a fixed linear diagnostic sequence.

Collectively, multimodal imaging is moving cardiac tumor diagnosis toward greater precision, integration, and clinical relevance. Echocardiography remains the gatekeeper for first-line assessment, whereas CMR, CT, and PET-based imaging provide complementary tissue, anatomic, and metabolic information when greater diagnostic certainty is required. Emerging AI-based fusion strategies may further strengthen this pathway in the future, although widespread clinical adoption will depend on improved standardization, reproducibility, and cross-modality harmonization (8, 62) (Figure 4).

## 7 Conclusion

Multimodal echocardiographic techniques have become an important component of cardiac tumor imaging. The evolution from purely structural assessment to quantitative characterization, perfusion analysis, functional integration, and AI-assisted interpretation has expanded the role of echocardiography in lesion localization, phenotypic characterization, and preoperative planning. Three-dimensional echocardiography, CEUS, and TDI/STE provide complementary information beyond conventional two-dimensional imaging, while AI and radiomics are increasingly being explored as supportive tools for workflow standardization and quantitative analysis, with expert oversight remaining essential (8, 10, 11, 48).

Nevertheless, important challenges remain. Current multimodal imaging approaches still lack standardized acquisition protocols and harmonized post-processing workflows across devices and centers; the operator-dependent nature of ultrasound limits quantitative consistency; and robust evidence from large multicenter, cross-vendor studies remains limited. In addition, true multimodal fusion is still at an early stage, and methodological frameworks for cross-modal alignment and feature integration across structural, perfusion, and metabolic domains have yet to mature fully (53, 55, 60).

Future efforts should focus on:

1. establishing standardized, cross-device and cross-center quantitative systems to unify measurement and reporting of CEUS, 3D echocardiography, and TDI/STE;
2. advancing AI, radiomics, and interpretable multimodal models to improve reproducibility and generalizability; and
3. developing integrated risk-prediction frameworks that combine clinical, imaging, and metabolic biomarkers to support personalized treatment planning and follow-up (9, 61, 67, 68).

Overall, multimodal echocardiography is likely to remain a key gatekeeper within a broader multimodality imaging ecosystem for cardiac tumors. Its greatest clinical value lies in first-line detection, dynamic assessment, and triage, while CMR, CT, and PET-based imaging provide complementary tissue, anatomic, and metabolic information when greater diagnostic certainty is required. Continued progress in standardization, algorithm robustness, and high-quality multicenter validation will be essential for successful clinical translation.
